# Effects of early preventive dental visits and its associations with dental caries experience: a cross-sectional study

**DOI:** 10.1186/s12903-022-02190-6

**Published:** 2022-04-29

**Authors:** Xing Qu, Shannon H. Houser, Meirong Tian, Qiong Zhang, Jay Pan, Wei Zhang

**Affiliations:** 1 Institute of Hospital Management, West China Hospital, Sichuan University, No. 37, Guoxue Lane, Chengdu, 610041 China; 2 Department of Health Services Administration, University of Alabama at Birmingham, Birmingham, AL 35294 USA; 3 Department of Paediatric Dentistry, West China Hospital of Stomatology, State Key Laboratory of Oral Diseases and National Clinical Research, Sichuan University, Sichuan University, Chengdu, 610041 China; 4 HEOA Group, West China School of Public Health and West China Fourth Hospital, Sichuan University, Chengdu, China; 5 Institute for Healthy Cities and West China Research Center for Rural Health Development, Sichuan University, Chengdu, China; 6 West China Biomedical Big Data Center, West China Hospital, Sichuan University, Chengdu, 610041 China; 7 Med-X Center for Informatics, Sichuan University, Chengdu, 610041 China

**Keywords:** Dental Caries, Office Visits, Dental Care for Children, Dental Health Services, Primary Prevention

## Abstract

**Objectives:**

Limited information is known about preventive dental visits (PDVs) before seven years of age among children in China. This study aimed to examine the early PDV rate, identify the impact of PDV on dental caries and untreated dental caries, and explore the factors related to PDV among Chinese sampled children under seven years old.

**Methods:**

A cross-sectional survey was conducted in five selected primary health care facilities in Chengdu, China, from May to August 2021. Parent–child dyads during regular systematic medical management were recruited to participate. Children's dental caries were identified through dental examinations and documented as decayed, missing and filled teeth index (dmft) by trained primary care physicians. Dental-related information was collected through a questionnaire. Zero-inflated negative binomial (ZINB) regression was used to test the effect of early PDV on the dmft value, and logistic regression was used to analyse impact factors on the early PDV.

**Results:**

A total of 2028 out of 2377 parent–child dyads were qualified for analysis. Half of the children (50.4%) were male, with a mean age of 4.8 years. Among all the children, 12.1% had their first dental visit for preventive purposes, 34.4% had their first dental visit for symptomatic purposes, and more than half had never visited a dentist. The results showed that a lower dmft value (adjusted OR: 0.69, 95% CI: 0.48–0.84), a higher rate of caries-free (aOR: 6.5, 95% CI: 3.93–10.58), and a lower rate of untreated dental caries (aOR: 0.40, 95% CI: 0.21–0.76) were associated with early PDV utilization. Children who had a higher rate of PDV were positively associated with living in a family with better parental behaviours (aOR: 2.30, 95% CI: 1.71–3.08), better parental oral health perception (aOR: 1.23, 95% CI: 1.06–1.32), fathers who had no untreated caries (aOR: 0.68, 95% CI: 0.47–0.97), families with higher socioeconomic status (aOR: 1.09, 95% CI: 1.04–1.16), and dental health advice received from well-child care physicians (aOR: 1.47, 95% CI: 1.08–2.00).

**Conclusions:**

Early PDV was associated with a lower rate of dental caries prevalence and untreated dental caries among sampled children younger than seven in Western China. Underutilization and social inequities existed in PDV utilization. Public health strategies should be developed to increase preventive dental visits and eliminate social disparities that prevent dental care utilization.

**Supplementary Information:**

The online version contains supplementary material available at 10.1186/s12903-022-02190-6.

## Introduction

Dental caries is one of the most common chronic diseases among children [1]. Untreated caries in deciduous teeth is the 10th-most prevalent condition, affecting 621 million children worldwide, especially in low-income families [2, 3]. Multiple risk factors were associated with early childhood caries (ECC), such as a high number of cariogenic oral bacteria, frequent intake of high sugar food, insufficient saliva flow, lower fluoride exposure, poor oral hygiene, and low socioeconomic demographic status [4]. Therefore, use of comprehensive methods for ECC prevention may be more effective.

The specific preventive interventions could be categorized into passive and active interventions [5]. A well-known passive preventive intervention is community fluoridated water. Compared to passive prevention, active preventive intervention requires a person to implement actions to change their lifestyle by including or excluding living habits [6]. Early preventive dental visits (PDVs) are one of the most critical active preventive interventions. Many dental professional academics recommend that parents take their children to the dental office when the first tooth erupts or no later than one year old [7, 8]. Early PDVs can provide more effective and less costly dental care than dental care provided in emergency care facilities or hospitals [9, 10]. However, there are significant disparities in access to early dental care among the population. The age of the first dental visit varies from one to six worldwide [11, 12]. Underutilization of early dental care was associated with ethnic minorities [13], lower family income [14], poorer parents’ perceptions and attitudes on dental health [15], less multidisciplinary cooperation [16], and lower access to dental services [17]. Understanding these factors is essential for developing strategies to eliminate disparities in early dental care utilization.

ECC remains a substantial public health issue in China, as in other countries in the world. According to the Fourth Chinese National Oral Health Survey, the prevalence of dental caries was 50.8%, 63.6%, and 71.9% for 3-, 4- and 5-year-olds, respectively [18]. Untreated dental caries were reported in 16.2% to 83.6% of different cities [19, 20]. Children’s oral health has not been effectively improved in recent decades [18]. In 2019, China’s National Committee of Health issued the Medium-to-Long Term Plan for the Prevention and Treatment of Chronic Diseases [21]. In this plan, early dental health management for children was proposed for the first time. There have been an increasing number of Chinese studies on children’s dental service utilization in recent years. All-cause dental care utilization among children was reported to range from 17.5 to 45.5% [22–24]. However, most of these studies indicated that more dental utilization was associated with a higher prevalence of dental caries. One reason to explain this was that those studies could not distinguish the time sequence between dental utilization and dental caries onset within a cross-sectional design. The other reason was that those studies did not differentiate between dental services for prevention and treatment purposes. This information is not good for supporting public health strategies targeting ECC.

This study aimed to investigate the current status of early PDV utilization among children under seven years old in western China. It seeks to address the following research questions: 1) what is the rate of early PDV among young children in a metropolitan city in Western China; 2) can early PDV play a positive role in reducing ECC and untreated dental caries; and 3) what family background characteristics are associated with PDV?

## Methods

### Study participants

A cross-sectional survey was conducted in the selected primary care settings in Chengdu, China, from May 2021 to August 2021. Following the judgemental sampling method, we selected primary care facilities that cover at least 80 thousand persons per facility (average cover scope is approximately 30 to 50 thousand) in the serving area and has two or more paediatric primary care physicians (PCPs) working in the facility [25]. Nine facilities satisfied the selection criteria, and five facility directors agreed to participate. Parent–child dyads were invited to participate by fifteen trained PCPs during regular systematic medical management. Children under seven years old who had no systemic disease were eligible for the study. Children’s parents or other caregivers who refused to participate were excluded from the study.

The sample size was calculated via the formula $n=p(1-p){(\frac{Z}{E})}^{2}$, where *Z* is the value from the standard normal distribution reflecting the confidence level that will be used (*Z* = 1.96 for 95%) and *E* is the desired margin of error (*E* = 0.5). *P* is the proportion of caries rate referred from the fourth national oral health survey [18]. The value of *p* was 0.508. Therefore, the sample size should be 385 in each facility. The minimum total sample size was 385 $\times$5 facilities = 1925. Considering a 10% error rate, the total sample size should be 2118.

During the systematic examination, children’s parents or other caregivers were invited to enroll in the survey by PCP in the office. When they agreed, they were asked to scan a QR code to electronically sign a consent form and subsequently complete an online questionnaire. The questionnaire included demographic status, parental oral health-related behaviours, and oral health status of the parents. Pretest and auto skip questions were set to avoid illogic errors in the questionnaire. Then, a dental examination was performed for the enrolled children.

### Primary outcome: dental caries and untreated dental caries

Children’s dental caries were measured by the decay/missing/filling teeth (dmft) index. Considering that dental caries were examined by PCPs, the decayed, missing and filled index (DMF) was used to identify evident caries. Compared to the International Caries Detection and Assessment System (ICDAS) and the Caries Assessment Spectrum and Treatment (CAST), the DMF may underestimate the occurrence of caries lesions in individuals but is the fastest method to apply [26]. Dental examination was performed and documented by the PCP during systematic medical management. For children under 18 months, the oral examination is conveniently performed in a “knee-to-knee” position. For children nearing two years of age, the PCP performed a dental exam with a flashlight for children who sat on the baby stool assisted by parents. For children over 30 months old, PCP performed dental examination with a flashlight with the child laid on the checking bed.

PCPs were required to perform a comprehensive evaluation for children following the guidelines of the National Basic Public Health Service for Children in China. This evaluation includes children’s physical development, vision health, and oral health. The PCPs who performed the evaluation should also give appropriate advice to parents. All the PCPs had studied basic oral health in college education and continuous education after they had graduated. In the present study, to strengthen PCP’s knowledge and update them on new paediatric dental knowledge, licenced paediatric dentists conducted two-hour face-to-face training for all participating PCPs [27]. The training content was about oral health knowledge and dental caries examination skills. The purpose of the training was to deepen their dental examination ability and ensure the accuracy of dental examinations. All PCPs passed a ten-question dental examination quiz after the training [28]. The average score was 81 out of 100. All single scores were above 70.

Untreated dental caries were collected both by examination and the questionnaire. If dmft > 0, parents were asked in the questionnaire, "Did you take your children to treat dental caries? (“Yes” or “No”). A child who had dental caries but took no treatment was defined as having untreated dental caries.

### Secondary outcomes: early preventive dental visits

Information on early preventive dental visits was collected using a self-report questionnaire. The first dental visit experience and age were collected by questions of "Did you ever take your child to visit a dentist?” (“Yes” or “No”.) If "Yes", “At what age did you take him or her to the first dental visit?”. We asked the question “Why do you take your child to the dental office at the first dental visit?” to assess the information for early preventive dental care. There are four options to answer this question: 1) had preventive care, e.g., regular checks, fluoride varnish, pit, and fissure sealant; 2) found a tooth decay onset; 3) the child reported a toothache or chewing issue; or 4) for other dental issues. If their parents or other caregivers chose option 1, we considered the children to have an early PDV. The age of PDV was used as the independent variable in the first step and the outcome variable in the second step. If they chose any other options from 2 to 4, we considered the children to have symptomatic dental visits. If they selected “no” dental visits in the first question, we considered the children to have no dental visits.

### Covariates

We adjusted selected covariates that were associated with PDV to explore more expected and unexpected factors. All covariates were selected according to previous studies. The covariates included children’s demographic information, parental oral health status, parent-performed dental hygiene behaviours, and family functioning.

Demographic information collected in this study included the child’s sex, age, family income level, living area, and distance from the dental clinic with paediatric dentistry. According to the Average Wage of Urban Workers of 2020 published by the Sichuan Provincial Bureau of Statistics [29], the average annual wage was Renminbi (RMB) 74,520. Converting RMB into US dollars was $11,291 per person, and the exchange rate was 6.6 in 2020. The average annual family income was estimated to be approximately $22,582 in families with two parents. In this study, we defined “low family income” as family income lower than the average level of $22,582; “middle family income” as equal to the upper two times the average level of $45,164; and “high family income” as family income higher than three times the average of $67,746. The family living area was measured as “urban” and “rural”. Dental clinic distance from home [30] was measured by driving time (within half an hour and more than half an hour).

Parental dental health perception and behaviours may impact the dental status and dental utilization of children [31, 32]. Parents’ caries status was measured by the following question: “Do any parent have dental caries but let it untreated? (Nobody, mother, father)”. The American Paediatric Dentistry Association recommended "cleaning baby's mouth and gums with a soft cloth or infant toothbrush at bath time before the teeth erupt, and parents should brush children teeth and supervise the brushing for school-age children until they are 7 to 8 years of age"[33]. We used the following two questions to represent parents’ dental care behaviour and perception. Dental care behaviour was represented by the question: "When did you start to brush or wipe gums/teeth for babies?” (“Before child one year old” or “After one year old”). “Before child one year old” represents better parental dental behaviour. Parental dental health perception was represented by the question: "At what age do you think your child could independently brush their teeth without parental supervision?" The options were 2–8 years. Older age indicates better parental dental perception and knowledge. Parents’ dental knowledge sources [24] were also collected (from the internet, books, friends, kindergarten teachers, and PCPs).

Family functioning includes partner relations, organization, and mother's education [34]. In this study, family functioning included the number of children per family (“one”, “two children”, or “more”), parental marital status (“married”, “divorced/separated”), and main caregivers of children (“parents”, “grandparents/babysitter”).

We also controlled for toothache status, which was reported to be an important indicator impacting dental health utilization among children in China [22]. The question was "Did your child have a toothache in the past six months? (“Yes” or “No”)”.

### Statistical methods

A three-step analysis was performed to achieve the study aims. First, descriptive information of the sampled population was summarized. Chi-square analysis was used to identify the difference in the proportion of PDV and the other service types in different subgroups. Second, to test the PDV effect on dmft, the zero-inflated Poisson model (ZIP) and zero-inflated negative binomial model (ZINB) [35] were employed because the dmfs index was not normally distributed (Kolmogorov–Smirnov test, *P* < 0.001) and was not fit for the condition of conventional regression models. The Vuong test [36] was used to determine whether estimating a zero-inflation component is more appropriate than the standard model. The variance inflation factor (VIF) was calculated to assess the multicollinearity across independent variables before ZIP and ZINB were performed. The Akaike information criterion (AIC) and the Bayesian information criterion (BIC) were used to report the model fitness, and the smaller the value was, the better. Then, logistic regression was used to test the PDV effect on untreated dental caries among the children who already had dental caries. Third, stepwise logistic regression was used to test the associations with PDV.

Statistical significance was set at 0.05. All analyses were performed by STATA 14.0 (Stata Corporation, College Station TX, USA).

## Results

### Descriptive information

A total of 2377 parent–child dyads were invited to the survey. A total of 135 children older than seven years and 214 parents who did not complete the questionnaire were excluded as missing completely at random. Finally, 2028 (70.5%, 2028/2877) children were included for analysis. Among the total population (Table 1), the mean age of the children was 4.8 years (SD: 1.18), 50.4% (N = 1023) were male, and 39.9% (809) had siblings. Of the total population, 42.2% (855) of children had dental caries, the mean dmft was 1.51, the range was 0–17, and 18.1% (368) had toothache experience within six months. A total of 12.1% (245) of children had their first dental visit for prevention, 34.4% (698) of children had their first dental visit for treatment, and 53.5% had never visited a dentist (*P* < 0.01). The mean age was 5.3 years for SDV and 4.8 years for PDV. Only 1.1% (23) of children had PDV before three years of age.

**Table 1:** Descriptive information of total sampled individuals divided by the first dental service utilization patterns

	Total	Symptomatic user	Preventive user	No visit	*P* value
	N (%)	N (%)	N (%)	N (%)	
Total	2028 (100)	698 (34.4)	245 (12.1)	1085 (53.5)	
Sex					
Male	1023 (50.4)	355 (50.9)	132 (53.9)	536 (49.4)	0.433
Female	1005 (49.6)	343 (49.1)	113 (46.1)	549 (50.6)	
Age					
< 2	68 (3.4)	9 (1.3)	6 (2.4)	53 (4.9)	**< 0.001**
2 ≤ y < 3	110 (5.4)	14 (2.0)	17 (6.9)	79 (7.3)	
3 ≤ y < 4	631 (31.1)	129 (18.5)	84 (34.3)	418 (38.5)	
4 ≤ y < 5	587 (28.9)	218 (31.2)	69 (28.2)	300 (27.6)	
5 ≤ y < 6	563 (27.8)	282 (40.4)	62 (25.3)	219 (20.2)	
6 ≤ y < 7	69 (3.4)	46 (6.6)	7 (2.9	16 (1.5)	
Mean age (SD)	4.8 (1.18)	5.3 (0.96)	4.8 (1.09)	4.5 (1.23)	
Children count					
One child	1219 (60.1)	414 (59.3)	165 (67.3)	640 (59.0)	**< 0.05**
Two or more children	809 (39.9)	284 (40.7)	80 (32.7)	445 (41.0)	
Dental caries					
No	1173 (57.8)	153 (21.9)	185 (75.5)	835 (77.0)	**< 0.001**
Yes	855 (42.2)	545 (78.1)	60 (24.5)	250 (23.0)	
Mean dmft	1.51(2.7)	3.14(3.4)	0.71(1.8)	0.63(1.6)	**< 0.001**
Education level of mother					
High school or blew	524 (25.8)	178 (25.5)	33 (13.5)	313 (28.8)	**< 0.001**
College	1364 (67.3)	473 (67.8)	179 (73.1)	712 (65.6)	
Master or above	140 (6.9)	47 (6.7)	33 (13.5)	60 (5.5)	
Family income category					
Low	637 (31.4)	233 (33.4)	53 (21.6)	351 (32.4)	**< 0.001**
Middle	915 (45.1)	304 (43.6)	102 (41.6)	509 (46.9)	
High	476 (23.5)	161 (23.1)	90 (36.7)	225 (20.7)	
Child’s main caregiver					
Parents	1840 (90.7)	638 (91.4)	230 (93.9)	972 (89.6)	0.236
Grandparents or others	188 (9.3)	60 (8.6)	15 (6.1)	113 (10.4)	
Parental marital status					
Normal	1954 (96.4)	671 (96.1)	236 (96.3)	1047 (96.5)	0.994
Divorce or separate	74 (3.6)	27 (3.9)	9 (3.7)	38 (3.5)	
Living area					
Urban	1955 (96.4)	681 (97.6)	240 (98.0)	1034 (95.3)	**< 0.05**
Rural	73 (3.6)	17 (2.4)	5 (2.0)	51 (4.7)	

The *P* value shows the difference in dental service utilization among subgroups

*dmft* decay/missing/filling teeth index

### Effect of PDV on dental caries level and untreated dental caries

The collinearity test results before the regression model showed that the VIF of the independent variables ranged from 1.02 to 1.31, indicating no collinearity. ZIP and ZINB were both performed. Since the Vuong test in ZIP was 12.74 and ZINB was 10.73, zero-inflated regression was more appropriate than linear regression for this dataset. According to the AIC and BIC (ZIP: 6057.9 and 6237.6, ZINB: 5694.5 and 5879.8) of the two models, ZINB fit the data better. Table 2 shows that preventive dental service utilization was significant in both parts of ZINB (p < 0.05), implying that a lower level of dental caries was among children who had more PDV (aOR: 0.60, 95% CI: 0.58–0.82) and more caries-free cases among children who had more PDV (aOR: 5.21, 95% CI: 3.59–7.57) after covariates were controlled.

**Table 2:** ZINB model and multivariable regression model of early preventive dental care effects on dental caries and untreated dental caries

	dmft		Untreated dental caries		
	ZINB model		Multivariable logistic regression model		
	(1)	(2)	(3)	(4)	(5)
	Negative Binomial part	Zero‐inflated part	Model 1	Model 2	Model 3
OR	0.69	5.21	0.41	0.34	0.40
95% CI	0.58–0.82	3.59–7.57	0.24–0.71	0.18–0.65	0.21–0.76
*P* value	< 0.01	< 0.01	< 0.01	< 0.01	< 0.01

ZINB model: Zero-inflated negative binomial model

Column (1) and (2) showed the effect of PDV on dmft. Zero‐inflated part showed a logit model for zero dmft (caries-free) children, predicting whether a child was in caries-free group. Negative binomial part showed dmft counts for those children who were not caries free. ZINB model was adjusted for children’s sex, age, family children count, age at which toothbrushing started, parents’ oral health perception for children, whether mother or father has untreated dental caries, children’s caregivers, rural or urban living area, parents’ marriage status, mother’s age and education, family income. Full table is in Additional file 1: S1

Column (3) Model 1: crude odds ratio indicating the effect of PDV on untreated dental caries. Column (4) Model 2: odds ratio adjusted by child’s sex, age, family children count, dmft, toothache, age at which toothbrushing started, age at first dental visit. Column (5) Model 3: odds ratio adjusted by Model 2 plus parents’ oral health perception for children, whether mother or father has untreated dental caries, children’s main caregivers, rural or urban living area, parents’ marriage status, mother’s age and education, and family income. Full table is in Additional file 1: S2

Among children who had dental caries, the association between untreated dental caries and PDV was estimated by multivariable logistic regression. As shown in column 3-4 in Table 2, Model 1 showed the crude odds ratio of the PDV effect on untreated dental caries. Then, Model 2 added covariates of child sex, age, family child count, dmft, toothache, age at toothbrushing start, and age at first dental visit plus Model 1. Model 3 was the final model containing all covariates of parents’ perception of age at supervised toothbrushing, whether mother or father has untreated dental caries, children’s main caregivers, rural or urban living area, parents’ marital status, mother’s age and education, and family income, plus Model 2. Model 3 showed that preventive dental utilization was steadily associated with lower untreated dental caries (aOR: 0.4, 95% CI: 0.21–0.76).

### Factors associated with early preventive dental visits

Among all the children (Table 3), multivariable logistic regression results showed that children's father had no untreated caries (aOR: 0.7, 95% CI: 0.47–0.97), longer distance from the dental clinic (aOR: 4.8, 95% CI: 3.54–6.57), and dental health knowledge from well-child care physicians (aOR: 1.5, 95% CI: 1.08–2.00), were associated with more PDV, in addition to the child who was younger age, family with a single child, better parents' oral perception, and higher socioeconomic status. On the other hand, the relationship was not significant for children’s sex, caregivers, dental health information from the internet, books, friends, schoolteachers, or parental marriage.

**Table 3:** Adjusted odds ratio of multivariable logistic regression for associated factors of preventive dental visits

	OR_adjusted_ (95%CI)	*P*
Sex	0.96 (0.71–1.28)	0.773
Age	0.88 (0.78–1.00)	**< 0.05**
More children (Ref: single child)	0.72 (0.54–0.97)	**< 0.05**
Age at starting brushing gum or teeth by parents’ earlier than one year old (Ref: later than one)	2.30 (1.71–3.08)	**< 0.01**
Age at parental perception on supervised tooth brushing (Continuous variable)	1.23 (1.06–1.32)	**< 0.01**
Mother has untreated caries	1.16 (0.79–1.71)	0.459
Father has untreated caries	0.68 (0.47–0.97)	**< 0.05**
Grandparents as child caregiver (Ref: Parents)	0.66 (0.37–1.12)	0.121
Resource of oral health information (Ref: None of above)		
Internet	0.75 (0.55–1.02)	0.064
Book	1.26 (0.87–1.82)	0.217
Friends	1.03 (0.69–1.53)	0.891
Well-child physician	1.47 (1.08–2.00)	**< 0.05**
School teachers	0.85 (0.60–1.20)	0.354
Living in urban (Ref: rural)	1.27 (0.46–3.40)	0.633
More than half hour from dental clinics (Ref: Less or equal than half hour)	4.82 (3.54–6.57)	**< 0.01**
Parental divorced (Ref: normal)	1.21 (0.61–2.38)	0.591
Mother’s age	1.04 (1.00–1.08)	**< 0.05**
Education years of mother	1.31 (1.10–1.56)	**< 0.01**
Family income category	1.09 (1.04–1.16)	**< 0.01**
Constant	0.01 (0.00–0.07)	**< 0.01**

## Discussion

The primary finding of this study demonstrated that early PDV was associated with a lower rate of dental caries and untreated dental caries among children younger than seven in western China. This finding identified that early PDV was underutilized in the study sample. Social inequities existed in preventive dental care utilization. A lower rate of PDV was associated with children who lived in families with more children, poorer parental oral care behaviours, fathers with untreated caries, and lower socioeconomic levels.

This study identified that the rate of first dental visits for prevention purposes was 12.1% among all children younger than seven in Chengdu, China. The rate of preventive dental care utilization was 24.3% in Beijing [24], and the average all-cause dental utilization rate at the national level was 17.6% [22]. The difference could be explained by socioeconomic-related inequality in dental utilization in different places [37]. In addition, we also identified that a higher education level of the mother, higher family income [14], better parental oral health perception and behaviours [15], and single children in the family [38] were associated with a higher rate of PDV, which is consistent with earlier research. This finding proved that there were social inequalities in utilizing early preventive dental care in Western China. The children who lived in families with higher educated mothers and higher family income had a significantly higher rate of preventive dental care utilization in the present study, which was consistent with previous studies [39–41]. Many social determinants for health tend to cluster among individuals living in underprivileged conditions and interact with each other. According to the framework for tackling social determinants, health inequities [42] are important predictors of health, especially in developing countries. More comprehensive actions should be taken to eliminate the social disparities of dental care among young children. For example, clarify the mechanisms by which social determinants generate oral health inequities and provide a framework for evaluating which social determinants are the most important to address among children in different areas.

The findings revealed that health advice from PCP had a positive effect on children’s PDV, which is consistent with previous international studies [43]. This result highlighted the potential oral benefits of the almost full cover of the National Basic Health Service Program in China, which covered 92.7% of children under seven in 2018[44]. Dental implementation by PCPs has been reported to be rare in China until now. More research should evaluate the advantages and cost-effectiveness of reducing caries by controlling common risk factors from the perspective of primary care. In addition, we unexpectedly found that higher PDV was associated with further living distance from dental clinics. Similar to patients who would like to seek orthodontics and dental implant services in further places to find a specific dentist [45], this unexpected result may reflect an extreme imbalance between paediatric dentistry supply and preventive dental demand. Parents who lived near the central city could access dental care knowledge through the Internet or social media development, yet a shortage of paediatric dentists broadly exists in China [46]. Children who live in suburban care may travel a long distance to access preventive dental care in the central city. Therefore, according to these results, we proposed that Chinese health policy-makers consider improving preventive dental visits by encouraging interdisciplinary cooperation between primary care and paediatric dentistry. However, in the long term, more paediatric dentists are needed to satisfy children’s dental care needs.

There are several limitations to this study. First, we could not observe continuous preventive dental service utilization in the present study, which is a better independent variable than the one-time variable [47]. Panel data should be collected to accurately evaluate the results of future studies. Second, hidden caries may be missing without mouth mirrors for examinations by PCPs, resulting in a lower number of dental caries. This may cause the association between dental caries and PDV to be underestimated. Third, sample selection bias was inevitable. The sample could partially represent the children who lived around the large primary care centre in the metropolitan city in western China. Besides, disparities between urban and rural areas could not be identified, and the differences may be larger. Fourth, we used a single parental report question to measure toothache, considering the time cost of responding, which may underestimate the actual proportion of children who suffer from toothache [48]. Future research is warranted to generalize our findings.

Despite these limitations, the present study has several contributions. The major strength of the present study is that it is the first study to find that early preventive dental utilization was associated with lower dental caries prevalence and lower untreated dental caries, which is inconsistent with some international studies [10, 49] and opposite to some domestic studies [18, 22]. The explanation is that we identified the time sequence and pattern of dental care services by using the first dental visit and its purpose as the independent variable. Dental caries can occur throughout life, both in primary and permanent dentitions [50]. Therefore, it is challenging to evaluate the real association between preventive dental care utilization and dental caries prevalence in a cross-sectional study. The advantage of using the first dental visit to identify the time sequence is that the first dental visit occurred previously or simultaneously with dental caries onset. The first dental visit is usually not interfered with by dentists’ examination but spontaneously performed relying on parents’ oral knowledge, attitude, and perception. Therefore, the purpose of the first dental visit could appropriately reflect parents’ active prevention perception. Notably, this variable could be better used in a survey targeting young children to reduce memory bias.

## Conclusions

Early PDV utilization was insufficient, and huge socioeconomic disparities existed among children. Preventive health policy strategies should focus on eliminating these disparities and increasing PDV access to promote dental health for young children. More longitudinal studies should evaluate the effect of PDV on dental health outcomes in China.

## Supplementary Information

**Additional file 1.** **S1.** ZIBN regression results of association between tooth decay and preventive dental visits among children. **S2.** Multivariable regression results of association between untreated dental caries and preventive dental visits among children who had dental caries.

## Data Availability

The datasets used and/or analysed during the current study are available from the corresponding author on reasonable request.
