# Supplementary material for: Effects of early preventive dental visits and its associations with dental caries experience: a cross-sectional study
Source: BMC Oral Health. 2022 Apr 29;22:150. doi: 10.1186/s12903-022-02190-6 (PMC9052678; doi:10.1186/s12903-022-02190-6)
Supplement: Supplementary file 1 — Additional file 1. S1. ZIBN regression results of association between tooth decay and preventive dental visits among children. S2. Multivariable regression results of association between untreated dental caries and preventive dental visits among children who had dental caries. [file 12903_2022_2190_MOESM1_ESM.docx]

Supplementary

S1 ZIBN regression results of association between tooth decay and preventive dental visits among children

|  |  | OR_adjusted_ | *P* value | 95% Confidence Interval | |
| --- | --- | --- | --- | --- | --- |
| **Negative Binomial part** | |  |  |  |  |
|  | Child number count | 1.12 | **<0.01** | 1.05 | 1.20 |
|  | Male (Ref: female) | 1.03 | 0.46 | 0.95 | 1.11 |
|  | Child’s age | 0.97 | 0.18 | 0.93 | 1.01 |
|  | Brushing gum or teeth with their parents’ assistance after one year old  (Ref: before one year old) | 1.06 | 0.13 | 0.98 | 1.15 |
|  | Better parental dental perception | 1.07 | **<0.01** | 1.03 | 1.10 |
|  | Mother untreated caries (Ref: No untreated caries) | 1.02 | 0.66 | 0.93 | 1.12 |
|  | Father untreated caries (Ref: No untreated caries) | 1.06 | 0.20 | 0.97 | 1.15 |
|  | Grandparents as main caregiver (Ref: parents) | 0.97 | 0.65 | 0.87 | 1.09 |
|  | Living at urban area (Ref: rural) | 1.08 | 0.51 | 0.87 | 1.34 |
|  | Parents’ divorce or live apart (Ref: Normal) | 0.63 | **<0.01** | 0.48 | 0.82 |
|  | Preventive dental visits | 0.69 | **<0.01** | 0.58 | 0.82 |
|  | (Ref: no visit and symptomatic dental visit) |  |  |  |  |
|  | Age at the first dental visit | 1.11 | **<0.01** | 1.08 | 1.14 |
|  | Mother’s age | 0.99 | **<0.01** | 0.98 | 1.00 |
|  | Education years of mother | 0.94 | **<0.01** | 0.89 | 0.98 |
|  | Family income category (Ref: lowest income) | 1.03 | **<0.01** | 1.01 | 1.04 |
|  | Constant | 5.37 | **<0.01** | 3.09 | 9.34 |
| **Zero‐inflated part** | |  |  |  |  |
|  | Child number count | 0.74 | **<0.01** | 0.60 | 0.92 |
|  | Male (Ref: female) | 1.02 | 0.86 | 0.82 | 1.27 |
|  | Child’s age | 0.82 | **<0.01** | 0.74 | 0.91 |
|  | Older child age of brushing gum or teeth with their parents’ assistance (Ref: before one year old) | 1.31 | 0.02 | 1.05 | 1.65 |
|  | Better parental dental perception | 0.96 | 0.39 | 0.88 | 1.05 |
|  | Mother untreated caries (Ref: No untreated caries) | 0.52 | **<0.01** | 0.39 | 0.70 |
|  | Father untreated caries (Ref: No untreated caries) | 0.75 | **<0.05** | 0.58 | 0.97 |
|  | Grandparents as main caregiver (Ref: parents) | 0.91 | 0.52 | 0.67 | 1.22 |
|  | Living at urban area (Ref: rural) | 1.41 | 0.26 | 0.78 | 2.56 |
|  | Parents’ divorce or live apart (Ref: Normal) | 0.83 | 0.56 | 0.45 | 1.54 |
|  | Preventive dental visits  (Ref: no visit and symptomatic dental visit) | 5.21 | **<0.01** | 3.59 | 7.57 |
|  | Age at the first dental visit | 0.56 | **<0.01** | 0.52 | 0.60 |
|  | Mother’s age | 1 | 0.84 | 0.98 | 1.03 |
|  | Education years of mother | 1.18 | **<0.05** | 1.03 | 1.35 |
|  | Family income category (Ref: lowest income) | 1.01 | 0.77 | -0.04 | 0.05 |
|  | Constant | 7.69 | **<0.05** | 0.57 | 3.51 |

ZINB model: Zero-inflated negative binomial model

Zero‐inflated part showed a logit model for zero dmft (caries-free) children, predicting whether a child was in caries-free group. Negative binomial part showed dmft counts for those children who were not caries free.

S2 Multivariable regression results of association between untreated dental caries and preventive dental visits among children who had dental caries

|  | Model 1 | | | | Model 2 | | | | Model 3 | | | |
| --- | --- | --- | --- | --- | --- | --- | --- | --- | --- | --- | --- | --- |
|  | OR_crude_ | *P* | 95% Confident interval | | OR_adjusted_ | P | 95% Confident interval | | OR_adjusted_ | P | 95% Confidence Interval | |
| Preventive dental visit | 0.41 | **<0.01** | 0.24 | 0.71 | 0.34 | **<0.01** | 0.18 | 0.65 | 0.40 | **<0.01** | 0.21 | 0.76 |
| Male (Ref: female) |  |  |  |  | 0.69 | **<0.05** | 0.48 | 0.98 | 0.63 | **<0.05** | 0.44 | 0.92 |
| Age |  |  |  |  | 0.91 | 0.348 | 0.75 | 1.11 | 0.89 | 0.25 | 0.73 | 1.09 |
| Child number count |  |  |  |  | 1.14 | **<0.05** | 0.84 | 1.56 | 1.1 | 0.55 | 0.80 | 1.53 |
| Decay teeth number |  |  |  |  | 0.84 | **<0.01** | 0.78 | 0.89 | 0.83 | **<0.01** | 0.78 | 0.89 |
| Toothache (Ref: no toothache) |  |  |  |  | 0.42 | **<0.01** | 0.29 | 0.60 | 0.4 | **<0.01** | 0.27 | 0.58 |
| Brushing gum or teeth with their parents’ assistance after one year old (Ref: before one year old) |  |  |  |  | 0.62 | **<0.05** | 0.43 | 0.91 | 0.66 | **<0.05** | 0.45 | 0.97 |
| Age at the first dental visit |  |  |  |  | 0.36 | **<0.01** | 0.30 | 0.43 | 0.35 | **<0.01** | -1.24 | 0.29 |
| Better parental dental perception |  |  |  |  |  |  |  |  | 0.87 | 0.067 | 0.75 | 1.01 |
| Mother untreated caries (Ref: No active caries) |  |  |  |  |  |  |  |  | 1.48 | 0.94 | 2.32 | 2.317 |
| Father untreated caries (Ref: No active caries) |  |  |  |  |  |  |  |  | 1.13 | 0.551 | 0.75 | 1.70 |
| Grandparents as child caregiver  (Ref: Parents) |  |  |  |  |  |  |  |  | 1.00 | 0.999 | 0.59 | 1.71 |
| Living at urban area (Ref: Rural) |  |  |  |  |  |  |  |  | 1.10 | 0.882 | 0.32 | 3.73 |
| Parents’ divorce (Ref: Normal) |  |  |  |  |  |  |  |  | 1.15 | 0.777 | 0.45 | 2.95 |
| Mother’s age |  |  |  |  |  |  |  |  | 0.99 | 0.821 | 0.95 | 1.04 |
| Education years of mother |  |  |  |  |  |  |  |  | 0.75 | **<0.05** | 0.60 | 0.94 |
| Family higher income category  (Ref: lowest income) |  |  |  |  |  |  |  |  | 0.96 | 0.319 | 0.89 | 1.04 |
| Constant | 0.34 | 0 | 1.224 | 1.623 | 777.06 | **<0.01** | 61.013 | 784.033 | 6.66 | **<0.01** | 51.85 | 11645.48 |
